# Hyperferritinemia Drives Risk: Unravelling the Association Between Ferritin Levels and Multi-Organ Dysfunction in Acute Febrile Illness: An Emergency Medicine Perspective

**DOI:** 10.7759/cureus.99737

**Published:** 2025-12-20

**Authors:** Neeraj Singla, Neha Sharma, Nalin Sharma, Mandip Bhatia, Navneet Sharma

**Affiliations:** 1 Internal Medicine, Postgraduate Institute of Medical Education and Research, Chandigarh, Chandigarh, IND

**Keywords:** acute febrile illness, ards (acute respiratory distress syndrome), dengue virus infection, high serum ferritin, tropical infections

## Abstract

Background: Hyperferritinemia has been increasingly recognized as a marker of systemic inflammation, immune dysregulation, and multi-organ failure. This study examines the association between vital signs, laboratory parameters (including serum ferritin), and clinical outcomes (discharge and mortality) among patients presenting with acute febrile illness and multi-organ dysfunction in a tropical emergency department.

Aim: To determine the association between serum ferritin levels and multi-organ failure in patients with acute febrile illness.

Methods: The study was conducted in the emergency department of a tertiary-level institute. Patients with acute febrile illness and multi-organ dysfunction without a definitive diagnosis were enrolled. All participants underwent routine blood investigations (hemogram, liver, and renal function tests), tropical serology (dengue, leptospirosis, malaria, chikungunya, and scrub typhus), and measurement of inflammatory markers, including C-reactive protein and serum ferritin.

Results. A total of 72 patients were included; 44 (61.1%) were male, and 28 (38.9%) were female. Mortality was notably higher in patients over 50 years, especially those above 60. Fever was the predominant symptom in 69 (95.8%) patients, while 16 (22.2%) patients exhibited bleeding manifestations.

Key factors that were significantly associated with mortality included Glasgow Coma Scale (GCS) score (p = 0.002), blood urea (p = 0.001), serum creatinine (p = 0.004), serum ferritin (p = 0.034), serum bilirubin (p = 0.019), and serum glutamic-oxaloacetic transaminase (SGOT) levels (p = 0.048). These findings indicate that patients with severe organ dysfunction have a higher risk of mortality. The effectiveness of transfusions in modifying outcomes remains uncertain.

Conclusions: Mortality was highest among older patients and those with multiple organ involvement, neurological symptoms, respiratory distress, infections, or hemodynamic instability. Elevated serum ferritin levels were strongly correlated with increased mortality, multi-organ dysfunction, and prolonged hospital stays, reinforcing their prognostic value. Complications such as encephalitis, shock, myocarditis, and acute respiratory distress syndrome (ARDS) significantly increased mortality rates. Duration of hospitalization was also a critical determinant of survival.

## Introduction

Acute febrile illness (AFI) is one of the leading causes of hospital admissions in tropical countries, particularly during the monsoon and post-monsoon seasons. These illnesses are attributed to a diverse array of pathogens, including *Plasmodium* species (malaria), *Leptospira* (leptospirosis), *Rickettsia* (scrub typhus), *Salmonella typhi*/*paratyphi* (enteric fever), and arboviruses such as Dengue and Chikungunya [1,2]. In many cases, these infections can progress to systemic inflammatory response syndrome (SIRS) and multi-organ dysfunction syndrome (MODS), resulting in significant morbidity and mortality in adult patients.

Early identification of biomarkers that reflect disease severity is crucial for guiding triage and management decisions. Serum ferritin, traditionally recognized for its role in iron metabolism, has recently garnered interest as a potential prognostic biomarker in systemic inflammation and sepsis [3]. Marked elevations in ferritin are observed in hyperinflammatory conditions, including hemophagocytic lymphohistiocytosis (HLH), adult-onset Still’s disease, septic shock, and severe viral infections [4].

Hyperferritinemia may reflect macrophage activation and cytokine storm, which are commonly observed in severe tropical infections. Notably, significantly elevated ferritin levels have been documented in severe dengue and scrub typhus, both of which are frequent causes of MODS in tropical regions [5,6]. Similarly, in leptospirosis and malaria, increased ferritin is associated with greater disease severity and poorer outcomes [7]. These findings highlight the potential of ferritin as a surrogate marker for disease burden and immune dysregulation in tropical febrile illnesses.

Despite this, there remains a paucity of data evaluating the relationship between serum ferritin levels and clinical outcomes in adult patients presenting with AFI and MODS in emergency departments during tropical seasons. Given the nonspecific nature of early symptoms in many tropical infections, ferritin could serve as a valuable adjunct in early risk stratification.

This study aims to explore the association of serum ferritin levels and disease severity in adult participants with acute febrile illness presenting with multi-organ dysfunction syndrome in a tropical medical emergency setting. The findings could enhance our understanding of the prognostic role of ferritin and potentially inform early intervention strategies.

## Materials and methods

This prospective observational study was conducted in the Medicine Emergency Department of a premier tertiary care institute, following approval from the Institutional Ethics Committee. The study focused on patients presenting with acute febrile illness (AFI) and multi-organ dysfunction syndrome (MODS).

Patient population

Patients aged over 12 years with acute febrile illness, defined as fever >38.3°C persisting for more than 48 hours and with onset within the preceding 14 days, were eligible for inclusion. Enrollment required the presence of at least two of the following clinical features: a) Cytopenias (platelet count <100,000/cu.mm or total leucocyte count <4,000/cu.mm), b) hepatomegaly and/or splenomegaly, c) lymphadenopathy, d) systemic signs (rash, edema), e) respiratory distress, f) encephalopathy.

All patients underwent a comprehensive diagnostic workup, including tropical serologies (dengue, malaria, scrub typhus, *Leptospira*, typhoid), serum ferritin levels, lipid profile, and bone marrow examination in patients who were alert or regained consciousness, indicated for the diagnosis of hemophagocytes, and the H score was calculated.

Hyperferritinemia was defined as ferritin levels greater than 500 ng/mL and measured by standard automated chemiluminescence immunoassays. The H score is a weighted scoring system based on nine variables, including fever greater than 38.3°C, cytopenias, splenomegaly, hypofibrinogenemia, hypertriglyceridemia, hyperferritinemia (greater than 500 ng/mL), hemophagocytosis in the bone marrow, hepatomegaly, and elevated transaminases. A detailed bilingual patient information sheet was provided, and written informed consent was obtained from all participants.

Outcomes

The primary outcome was the correlation of serum ferritin levels measured at admission with mortality in patients with undifferentiated acute febrile illness. Secondary outcomes included the incidence of unfavorable complications in patients with MODS due to tropical infections associated with hyperferritinemia.

Statistical analysis

All data were entered into Microsoft Excel (Redmond, USA) and analyzed using IBM Corp. Released 2018. IBM SPSS Statistics for Windows, Version 24. Armonk, NY: IBM Corp. Quantitative variables were summarized as mean ± standard deviation (SD), and normality was assessed using the Kolmogorov-Smirnov test (n>50). Categorical variables were compared between survivors and non-survivors using the chi-square test or Fisher’s exact test, as appropriate. For intergroup comparisons of laboratory parameters, either the independent two-sample t-test or the Mann-Whitney U test was employed, depending on data distribution. A p-value < 0.05 was considered statistically significant.

## Results

This study examined demographic distribution, clinical symptoms, comorbidities, laboratory parameters, and clinical outcomes among 72 patients with hyperferritinemia in sepsis patients with multi-organ dysfunction syndrome.

The mean age of patients was 42.75 years (range: 13-95 years). Most patients (27.4%) were in the 31-40 age group. Males constituted 44 (61.11%) of cases, while females were 28 (38.9999%). Among the clinical presentations, fever was present in 69 (95.8%), followed by respiratory distress and gastrointestinal symptoms in 35 (47.9%), myalgias in 31 (42.5%), bleeding manifestations in 16 (22.2%), urinary symptoms in 13 (17.8%), altered mental sensorium in 22 (30.1%), and joint pain and arthralgias in less than 5% of patients. In bleeding manifestations, major organ bleed was present in 15 (20.8%), mucosal bleed was present in 9 (12.5%), and petechiae was present in 5 (6.9%), which is highlighted in Table 1.

**Table 1:** Comparison of symptoms and co-morbidities between survivors and non-survivors in patients with acute febrile illness and ferritin elevation (n=72) (p<0.05 is considered significant) AMS: Acute Mountain Sickness, COPD: Chronic Obstructive Pulmonary Disease, CAD: Coronary Artery Disease, CLD: Chronic Liver Disease, CKD: Chronic Kidney Disease

	Survivors (61)84.72%	Non-survivors (11)15.27%	Chi-Square Test	p-value
Fever	58(95.1%)	11(100%)	0.565	0.452
Arthralgias	2(3.3%)	1(9.1%)	0.788	0.375
Rash	4(6.6%)	2(18.2%)	1.649	0.199
Bleeding Manifestations	14(23.0%)	2(18.2%)	0.123	0.726
Petechiae	4(6.6%)	1(9.1%)	0.093	0.761
Mucosal Bleed	6(9.8%)	3(27.3%)	2.591	0.108
Major Organ Bleed	13(21.3%)	2(18.2%)	0.055	0.814
Joint Pains	2(3.3%)	1(9.1%)	0.788	0.375
Myalgias	26(42.6%)	4(36.4%)	0.150	0.698
GI Symptoms	29(47.5%)	5(45.5%)	0.160	0.898
Urinary Symptoms	11(18.0%)	2(18.2%)	0.000	0.991
AMS	14(23.0%)	8(72.7%)	10.882	0.001
Respiratory Symptoms	25(41.0%)	9(81.8%)	6.235	0.013
Co-morbidities				
Diabetes Mellitus	10(16.4%)	2(18.2%)	0.021	0.884
Hypertension	11(18.0%)	2(18.2%)	0.000	0.991
COPD	1(1.6%)	3(27.3%)	11.671	0.001
CAD	1(1.6%)	1(9.1%)	1.872	0.171
CLD	0(0%)	0(0%)		
CKD	4(6.6%)	2(18.2%)	1.649	0.199

On clinical examination, the mean Glasgow Coma Scale (GCS) score was found to be 13.36. Systolic blood pressure (SBP) averaged 114.50 mmHg, while diastolic blood pressure (DBP) was 72.14 mmHg.

Mean hemoglobin (HB) was 12.15 g/dL, and total leukocyte count (TLC) averaged 12,086.25. Platelet count was highly variable, ranging from 1.19 to 226,000. Ferritin levels had a wide distribution, with a mean of 8,655.46, which has been illustrated in Table 2.

**Table 2:** Comparison of laboratory investigations and tropical serologies between survivors and non-survivors in patients with acute febrile illness and ferritin elevation (p<0.05 is considered significant) HB: Hemoglobin, TLC: Total Leukocyte Count, HCT: Hematocrit, CRP: C-Reactive Protein, SGOT: Serum Glutamic Oxaloacetic Transaminase (AST), SGPT: Serum Glutamic Pyruvic Transaminase (ALT), ALP: Alkaline Phosphatase

Lab investigations	Survivors		Non-Survivors			
	Mean	Standard Deviation	Mean	Standard Deviation	T-value Mann-Whitney test	p-value
HB	12.24	2.83	11.66	1.84	.653	.516
TLC	12041.15	17650.38	12336.36	7702.89	1.127	0.26
Platelets	33391.72	40664.91	43818.18	26301.40	1.722	0.085
Neutrophils	62.24	13.94	68.36	16.66	1.301	.198
Lymphocytes	23.54	12.62	18.59	15.95	1.150	.254
HCT	37.87	8.14	36.42	8.33	0.541	.590
CRP	70.70	68.80	93.71	66.26	0.839	.405
Ferritin	7963.27	17821.86	12654.78	19376.25	2.115	.034*
Triglycerides (TG)	277.76	173.01	265.40	212.55	0.148	.883
Fibrinogen	3.01	1.51	2.74	2.52	0.366	.716
H Score	140.89	55.35	143.60	57.13	0.102	.919
Urea	68.96	66.92	135.09	74.64	3.194	.001**
Creatinine	1.66	1.76	3.09	1.77	2.85	.004**
Sodium (Na)	135.33	7.62	138.55	6.31	1.319	.192
Potassium(K)	4.37	.66	4.58	.75	0.935	.353
Bilrubin	2.30	3.13	4.84	6.87	1.698	0.089
SGOT	991.74	2141.78	1425.55	1889.93	2.34	.019*
SGPT	566.43	1297.26	589.45	722.82	1.980	.048*
ALP	191.68	159.73	241.64	117.32	1.857	0.063
Albumin	2.96	.71	2.68	.66	1.236	.221
Tropical Serology					Chi-square test	
Dengue	45(73.77%)		8(72.7%)		0.123	0.739
Scrub Typhus	11(18.03%)		3(27.3%)		0.395	0.53
Leptospirosis	6(9.8%)		1(9.1%)		0.016	0.96
Chikungunya	5(8.19%)		1(9.1%)		0.003	0.96
Enteric Fever	0		0			
Malaria	0		0			
No Specific Diagnosis	5(8.19%)		0		1.224	0.269

Dengue fever was the most common infectious cause (76.8%), followed by scrub typhus (20.3%) and leptospirosis (10.1%). Malaria and enteric fever were not reported. Blood, urine, or pus cultures were positive in 13.9% of cases, with a significantly higher prevalence among non-survivors (p=0.019).

Amongst radio-imaging abnormalities, pleural effusion was observed in 38.4% of cases, ascites in 45.2%, and hepatomegaly in 30.1%. Splenomegaly was noted in 13.7% of cases. Imaging abnormalities, such as bilateral infiltrates, were found in 4.1% of patients. Dengue encephalitis was diagnosed in 1.4% of cases. Chest infiltrates, pleural effusion, and splenomegaly were more frequent in non-survivors.

Amongst the complications, serositis, bleeding manifestations, renal failure, encephalitis, shock, hepatitis, and myocarditis were seen in both survivors and non-survivors, showing a strong correlation with mortality, which is charted in Table 3.

**Table 3:** Comparison of complications between survivors and non-survivors in patients with acute febrile illness and ferritin elevation (p<0.05 is considered significant) ARDS: Acute Respiratory Distress Syndrome

	Survivors: 61(84.72%)	Non-Survivors :11(15.27%)	Chi-Square Test	p-value
ARDS	28(45.9%)	8(72.72%)	3.997	0.046
Severe Bleeding	15(24.59%)	1(9.09%)	1.048	0.306
Renal Failure	24(39.3%)	7(63.63%)	3.282	0.07
Encephalitis	11(18.03%)	7(63.63%)	12.26	0.0001
Shock	12(19.67%)	6(54.5%)	7.383	0.007
Hepatitis	46(75.4%)	8(72.72%)	0.099	0.753
Myocarditis	11(18.03%)	6(54.5%)	8.309	0.004
Coagulopathy	8(13.11%)	4(36.3%)	4.422	0.035
Musculoskeletal Manifestations	5(8.19%)	0	0.882	0.348
>1 Complication	40(65.6%)	7(63.6%)	0.675	0.784
Duration of Hospital Stay			6.189	0.045
≤ 5 days	24(39.3%)	5(45.45%)		
6-10 days	28(45.9%)	3(27.27%)		
>10 days	9(14.7%)	3(27.27%)		
Random Donor Platelet Transfusion	26(42.6%)	3(27.3%)	0.913	0.339
Single Donor Platelet Transfusion	22(36.1%)	2(18.2%)	1.341	0.247

In outcomes, 61 (84.7%) patients recovered, whereas 11 (15.3%) patients expired. During the hospital stay, <=5 days was seen in 29 (46.8%), with the highest mortality (p=0.045). 

A total of 6-10 days in 28 (45.2%), and more than 10 days' stay was seen in 5 (8.1%) patients, with no recorded deaths, suggesting longer treatment duration may have improved survival.

Transfusion therapies, including random donor platelets (RDP) and single donor platelets (SDP), were administered to 29 (40.3%) and 24 (33.3%) of patients, respectively, but RDP and SDP showed no significant impact on survival, p=0.339 and p=0.247, respectively. Steroid and intravenous immunoglobulin (IVIG) use was minimal, each administered to only 1.4% of patients.

Patients who were discharged had a higher mean Glasgow Coma Scale (GCS) score (13.87) compared to those who died (10.55, p=0.002). Systolic blood pressure (SBP) was lower in deceased patients (103.64 mmHg) than in discharged ones (116.46 mmHg). Similarly, diastolic blood pressure (DBP) was reduced in non-survivors (66.36 mmHg vs. 73.18 mmHg). Non-survivors had a higher mean pulse rate (102.09 bpm) than discharged patients (97.20 bpm), while oxygen saturation (SpO2) levels were slightly higher in non-survivors (95.82%) compared to survivors (92.84%).

Renal and hepatic function

Non-survivors exhibited significantly higher blood urea levels (135.09 vs. 68.96, p=0.001) and creatinine levels (3.09 vs. 1.66, p=0.004), indicating worsening kidney function. Serum sodium (Na) was slightly higher in non-survivors (138.55 vs. 135.33), while potassium (K) levels were comparable. Bilirubin was elevated in deceased patients (4.84 vs. 2.30, p=0.019), and liver enzyme levels (SGOT: 1425.55 vs. 991.74, p=0.048; SGPT: 589.45 vs. 566.43) were higher in non-survivors, indicating hepatic dysfunction.

**Figure 1:**
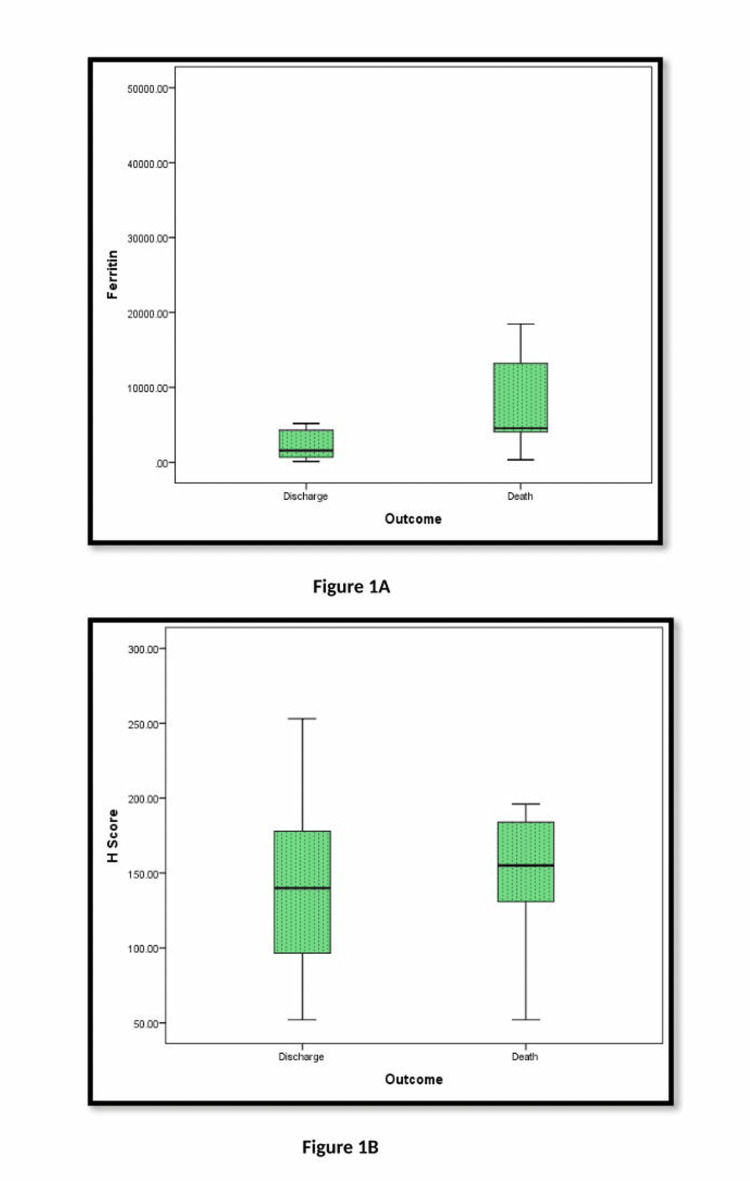
Box plot representation of the association of ferritin and HLH score with the patient’s outcome (discharge vs. death) 1 A. Ferritin vs. Outcome: the median ferritin level is higher in the "Death" group compared to the "Discharge" group, which suggests that elevated ferritin is associated with a worse outcome or disease severity. (p=0.0001**) 1 B. H Score vs. Outcome: A higher H score in the "Death" group suggests that patients with higher H scores are at greater risk of mortality, as the H score, often used to assess hyperinflammation, could be a prognostic marker. (p=0.819), highlighted the association between the final outcomes and both Serum ferritin, H-score.

Association of serum ferritin was assessed by Spearman-Pearson correlation with various clinical signs and laboratory parameters depicted in Table 4, and was found significant with H score (0.089), sodium levels (0.044), hemoglobin levels (0.084), potassium levels (0.012), ALT (0.021), and AST (0.000). Box plot analysis, as shown in Figure 1, highlighted the association between the final outcomes and both Serum ferritin and H-score.

**Table 4:** Association between various clinical parameters and laboratory investigations with serum ferritin (p<0.05 is considered significant) *. Correlation is significant at the 0.05 level (2-tailed); **. Correlation is significant at the 0.01 level (2-tailed) GCS: Glasgow Coma Scale, SBP: Systolic Blood Pressure, DBP: Diastolic Blood Pressure, SpO₂: Peripheral Capillary Oxygen Saturation, HB: Hemoglobin, TLC: Total Leukocyte Count, HCT: Hematocrit, CRP: C-Reactive Protein, TG: Triglycerides, Na: Sodium, K: Potassium, SGOT: Serum Glutamic Oxaloacetic Transaminase (AST), SGPT: Serum Glutamic Pyruvic Transaminase (ALT), ALP: Alkaline Phosphatase

	Ferritin	
	Pearson Correlation	p-value
GCS	-.005	.972
SBP	-.027	.839
DBP	-.006	.966
Pulse	.066	.614
Spo2	.130	.318
HB	.223	.084
TLC	.140	.281
PLATELETS	-.106	.416
NEUTROPHILS	-.015	.906
LYMPHOCYTES	-.149	.253
HCT	.167	.197
CRP	-.029	.830
TG	.053	.731
Fibrinogen	-.237	.113
H Score	.273	.089
UREA	.023	.859
Creat	.153	.240
Na	-.259^*^	.044
K	.321^*^	.012
BILRUBIN	-.026	.840
SGOT	.435^**^	.000
SGPT	.296^*^	.021
ALP	.016	.906
Albumin	-.007	.956
Duration of Hospital Stay	.085	.536

## Discussion

This study evaluates the prognostic significance of serum ferritin in predicting the severity and outcomes of patients with acute febrile illness and MODS. The findings align with previous research that highlights ferritin as an essential biomarker in critical illnesses such as sepsis, dengue, hemophagocytic lymphohistiocytosis (HLH), and macrophage activation syndrome (MAS) [4,8]. The study establishes that higher ferritin levels are correlated with increased mortality, multi-organ dysfunction, and prolonged hospital stays, thereby reinforcing their prognostic value.

Demographic comparisons

The cohort comprised 72 patients, with a mean age of 42.75 years, and predominantly in the 31-40 age group. Males constituted 61.1% of cases. These demographic trends have been observed in tropical illness-related MODS studies, where middle-aged adults, particularly males, have a higher risk of complications [9]. The predominance of dengue fever as the underlying etiology mirrors patterns observed in endemic regions, where dengue-associated MODS frequently affects younger adults [10].

Clinical profile and comparison

Fever was the most common symptom, reported in 95.8% of patients, consistent with tropical illness-related MODS studies where fever is a primary presenting feature [11]. Other common symptoms included myalgias (42.5%), gastrointestinal symptoms (47.9%), and respiratory symptoms (47.9%). Altered mental status (30.1%) was also significantly associated with mortality (p=0.001).

A noteworthy variance from bacterial sepsis studies was the lower prevalence of hypotension at presentation, as reflected in the mean systolic (114.5 mmHg) and diastolic (72.14 mmHg) blood pressures in this cohort. In contrast, sepsis-associated MODS often presents with profound hypotension and higher shock incidence [12]. However, shock remained a strong predictor of mortality in this cohort (p = 0.007), consistent with the global critical care literature.

Laboratory investigations and pathophysiology

Hyperferritinemia was strongly associated with disease severity. The mean ferritin level was 8,655.46 ng/mL, with significantly higher levels in non-survivors (12,654.78 ng/mL) compared to survivors (7,963.27 ng/mL, p=0.034). This trend is comparable to HLH-associated MODS, where ferritin levels exceeding 10,000 ng/mL are linked to increased mortality [13]. Elevated ferritin levels in this study suggest excessive inflammation, macrophage activation, and iron-mediated oxidative stress, similar to findings in cytokine storm syndromes [14].

Complications and multi-organ failure

MODS was associated with several complications, including respiratory failure (ARDS), neurological dysfunction, and shock. Encephalitis (p=0.0001) and ARDS (p=0.046) were significantly linked to mortality, consistent with existing research on systemic inflammation-driven organ failure [15].

Coagulopathy was pointedly more in non-survivors (40% vs. 13.1%, p=0.035), mirroring trends in disseminated intravascular coagulation (DIC) studies where hyper-ferritinemia contributes to coagulopathy and thrombocytopenia [16].

An Indian study guided by Suresh SC et al. [17] found that serum ferritin levels were significantly higher in patients with severe dengue, with a cutoff value of 714 ng/ml, compared to those with mild dengue, with a sensitivity of 100% and a specificity of 43.75%. which was in concordance with this study. Similarly, in a study guided by Kurian SJ et al. [18], median ferritin levels were significantly higher in moderate to severe COVID-19 infection compared to mild (p = 0.001). Both these studies were in concordance with the present study, which re-emphasized the higher serum ferritin values with more severity of illness.

Limitations

It was a single-center study, which limited its generalizability. There was a lack of serial ferritin measurements to assess temporal trends. No adjustment was made for the effects of iron supplements or blood transfusions on ferritin. The non-survivor group (n=11) was very small in this study. 

## Conclusions

This study reinforces the role of ferritin as a biomarker for assessing the severity of multi-organ dysfunction syndrome in patients with acute febrile illness. Given its strong association with systemic inflammation and organ failure, early ferritin measurement could help stratify patients based on risk, allowing for timely interventions such as immunomodulatory therapy. The findings indicate that neurological deterioration, renal impairment, elevated inflammatory markers, and hepatic dysfunction are significant contributors to mortality in ferritin-related conditions. Higher ferritin and C-reactive protein (CRP) levels were indicative of a more severe inflammatory state, and patients with pronounced metabolic and organ dysfunction exhibited poorer prognoses. Notably, dengue fever emerged as the most prevalent infectious etiology associated with hyperferritinemia in this cohort.

Effective management strategies should prioritize early detection, rigorous infection control, and comprehensive organ support to improve survival outcomes in patients with ferritin-related multi-organ dysfunction. We recommend measuring serum ferritin within 24 hours of admission in all acute febrile illness patients presenting with ≥2 organ dysfunctions.
